# CROSS-COUNTRY VALIDATION OF ANTHROPOMETRIC AGE (ANTHROPOAGE) AS A MEASURE OF BIOLOGICAL AGING

**DOI:** 10.1093/geroni/igad104.0948

**Published:** 2023-12-21

## Abstract

Aging occurs at unique rates at an individual and population-level. In contrast to chronological age (CA), biological age (BA) is better at capturing functional decline and risk of age-related diseases, disability, and death. Nonetheless, it is still unclear how this phenomenon changes across adults from different races/ethnicities. We recently developed a BA metric using anthropometric measurements (AnthropoAge) in participants from NHANES-III. Here, we aim to validate AnthropoAge across countries and to determine whether it displays population-specific patterns using harmonized cohorts from the U.S. and Mexico from the Gateway to Global Aging Data Team, including the Mexican Health and Aging Study and the Health and Retirement Study. We found that AnthropoAge is a robust predictor of overall mortality risk in both American (AUROC=0.797; 95%CI 0.786-0.808) and Mexican (0.776; 0.740-0.812) adults, and this was consistent across multiple cutoffs of time. Notably, we found that the prediction improved in participants aged 50-75 years in comparison to older adults aged 75-100 years. AnthropoAge was also associated with number of comorbidities and disability in activities of daily living independently of CA. Finally, we observed marked changes of AnthropoAge and its acceleration across the years in both countries, with a significant interaction with Mexican ethnicity (beta for interaction: 0.2034; p=5.94E-05). AnthropoAge is a robust and reproducible measure of BA, however, new studies are required to decipher the heterogeneity of anthropometric aging in specific ethnicities and age categories.

